# Pattern of secondary growth in monocot roots: unveiling longitudinal and cross-sectional variability

**DOI:** 10.1007/s00425-025-04744-8

**Published:** 2025-06-24

**Authors:** Jan Marcinkiewicz, Joanna Jura-Morawiec

**Affiliations:** Polish Academy of Sciences Botanical Garden-CBDC in Powsin, Prawdziwka 2, 02-973 Warsaw, Poland

**Keywords:** Anatomy, *Dracaena*, Eccentric growth, Monocot cambium, Reaction wood, Vascular bundles

## Abstract

**Main conclusion:**

Monocot cambium activity varies along the root axis and circumference, resulting in eccentric secondary growth. Variation in secondary growth structure along the root diameter indicates functional specialization but without reaction wood characteristics.

**Abstract:**

Secondary growth in roots is one of the most important adaptive features, providing mechanical support to stabilize the aboveground part of an arborescent plant. Our knowledge of this phenomenon in arborescent monocots is limited: it occurs exclusively in *Dracaena* species, it has a bundled structure and it is formed by the monocot cambium. To add to our understanding, we investigated the pattern of secondary thickening along the axis and along the diameter of the stem-borne roots of a dragon tree *Dracaena draco* L. by analyzing the direction of eccentricity vector and examining root anatomy. We hypothesized that the distribution of secondary growth changes along the root and that there are anatomic differences between concentric secondary growth (uniform around the root) and eccentric secondary growth (asymmetric), that may shed light on its adaptive significance. We found that roots show irregular eccentricity, with the direction of the eccentricity vector changing from up to sideways, counterclockwise or clockwise. Vascular bundle density was lower in eccentric secondary growth and these bundles differed in size, shape, and components (tracheid lumen fraction, tracheid wall fraction) compared to concentric secondary growth. Distinct arcs in eccentric secondary growth were the result of varying thickness of the ground parenchyma cell walls, variation in bundle size, or a combination of both. Our study was a pioneering effort to investigate the variability of secondary growth along roots in monocots, and suggests a spatial separation of the mechanical and transport functions in the root, but without the contribution of features characteristic of reaction wood.

## Introduction

**Fig. 1:**
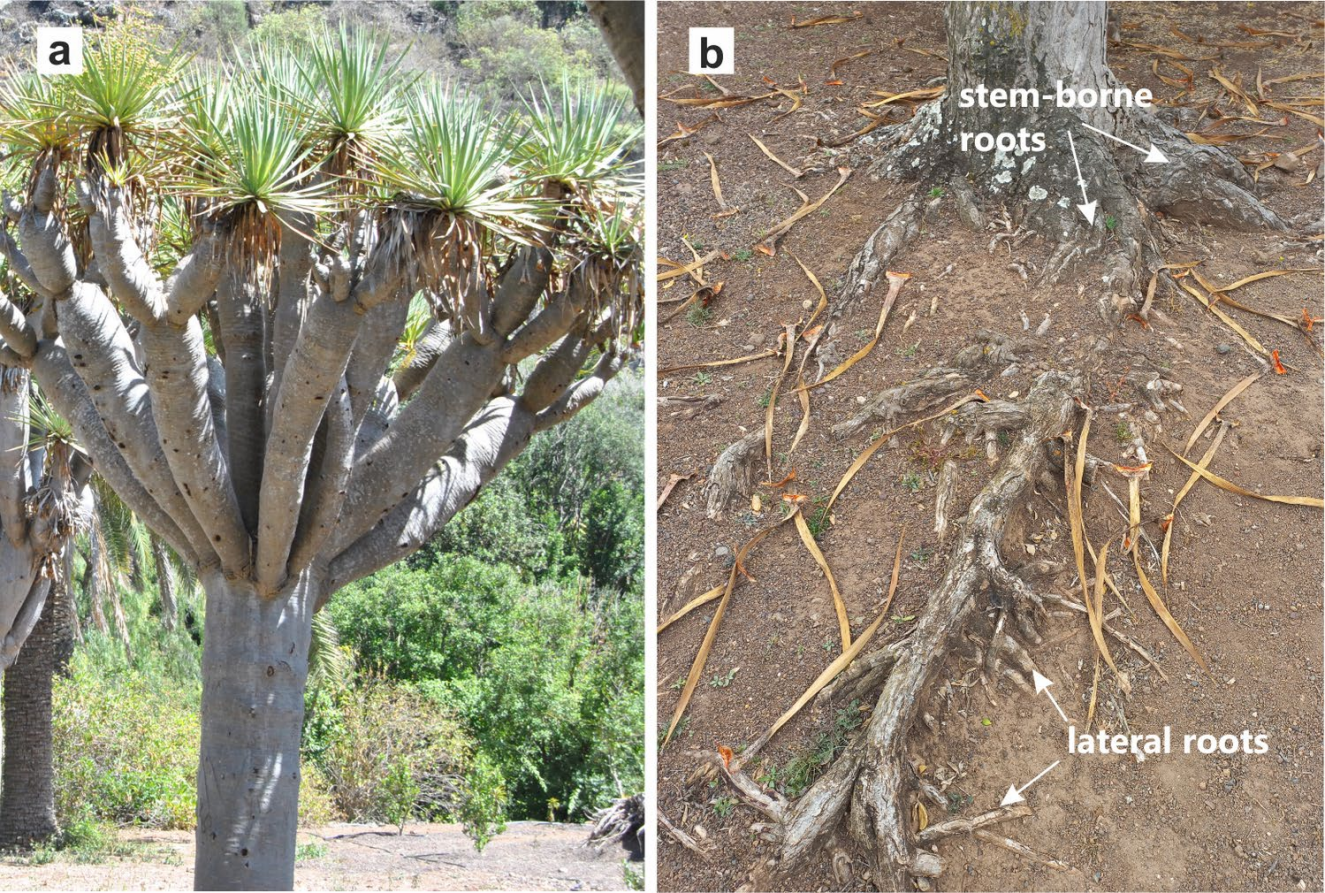
The dragon tree *Dracaena draco* and its root system. **a** Habit. **b** Stem-borne and lateral roots

Root anchorage characteristics vary between species. Most arborescent monocots, such as palms, yuccas and aloes, have a fibrous root system consisting of roots relatively homogenous in size and form. The exception among the monocotyledons are the species of *Dracaena* (Asparagaceae), the roots of which thicken with age due to secondary growth (Tomlinson and Zimmermann 1969; Kauff et al. 2000; Carlquist 2012). The tallest members of the genus *Dracaena*, known as dragon trees, develop a thick trunk and an underground root system that can extend well beyond the projection of the crown (Hubálková et al. 2017; Maděra et al. 2020). Their root system consists of the thickest stem-borne roots, their laterals and a network of thin fine roots (Jura-Morawiec et al. 2021; Fig. 1).

Secondary growth helps roots to adapt their shape, hydraulic and mechanical properties, to varying environmental conditions via cambial activity (Strock and Lynch 2020). Information on secondary growth in monocot roots is scarce. It is known to have a bundled structure, and to develop from the monocot cambium (Tomlinson and Zimmermann 1969; Carlquist 2012; Jura-Morawiec et al. 2021). The activity of the monocot cambium at the root base is not uniform around the meristem circumference, but is restricted to specific sectors, resulting in eccentric secondary growth, with more secondary tissue on the upper side of the root cross section (Marcinkiewicz and Jura-Morawiec 2024). Variations in the activity of the monocot cambium are also apparent in a ring-like pattern in secondary growth, resembling the annual rings in the wood of many trees (Cheadle 1937; Fisher 1975; Jura-Morawiec 2015; Marcinkiewicz and Jura-Morawiec 2024).

Current knowledge on the structure–function relationships of eccentric secondary growth in monocots comes almost exclusively from studies of inclined stems of *Beaucarnea*, *Cordyline*, *Dracaena* and *Yucca* species, which produce more secondary tissue on the lower stem side, like in an inclined conifer stem (Fisher 1975; Krawczyszyn and Krawczyszyn 2016). However, this asymmetric growth is not associated with the typical characteristics of reaction wood, such as rounded and abnormally thickened and lignified tracheid walls (compression wood) or a gelatinous, unlignified G-layer in the cell walls of fibers (tension wood). As in monocots, the righting of displaced stems only occurs in regions of primary growth (Tomlinson and Zimmermann 1969), similar to Cycadales (Fisher and Marler 2006).

In the recent study (Marcinkiewicz and Jura-Morawiec 2024) we found that at the root base, it is possible to distinguish the area of concentric and eccentric secondary growth, based on the density of vascular bundles. Here, we continue this series, by describing a number of aspects of the secondary growth with increasing distance from the root base. We hypothesize that eccentric secondary growth is not uniformly distributed along roots, and there are anatomic differences between the concentric and eccentric secondary growth. Studying this structure may provide insights into its functional significance. Since secondary growth in roots plays a key role in adapting their shape and structure to changing conditions, the dynamics of factors influencing crown-stem-root-soil interactions, acting on the monocot cambium, should be reflected in the structure and distribution of secondary growth along the roots. Therefore, our aim was to study the structure and distribution of secondary growth in roots of dragon trees *Dracaena draco* L,. by analyzing the direction of the eccentricity vector in successive root disks and examining root anatomy in cross-sections.

## Material and methods

### Plant material

Stem-borne roots were collected from dragon trees *Dracaena draco* L. This species is native to the Canary Islands, Madeira and Morocco (Maděra et al. 2020) and is endangered according to the IUCN Red List. Sampling was restricted to roots of four plants with diameter at breast height (DBH) of 19, 20, 21 and 31 cm, one growing in the greenhouse of PAS BG-CBDC in Powsin, two in the commercial nursery (planted in Spain) and one growing outdoors in the Jardín Botánico Canario on the island of Gran Canaria, Spain. Sampling was restricted to four roots (one root per plant) because of the threat to plant survival from damage to the root system. It should be noted that research into secondary growth can only be carried out on roots from plants that are at least a few years old. Roots, 40–50 long to the first ramification, were cut at the base with a hand saw and, after marking the top, sliced into discs about 3.5–4-cm wide or at the points of interest, i.e., the zone of rapid tapering.

### Anatomy and microscopy

**Fig. 2:**
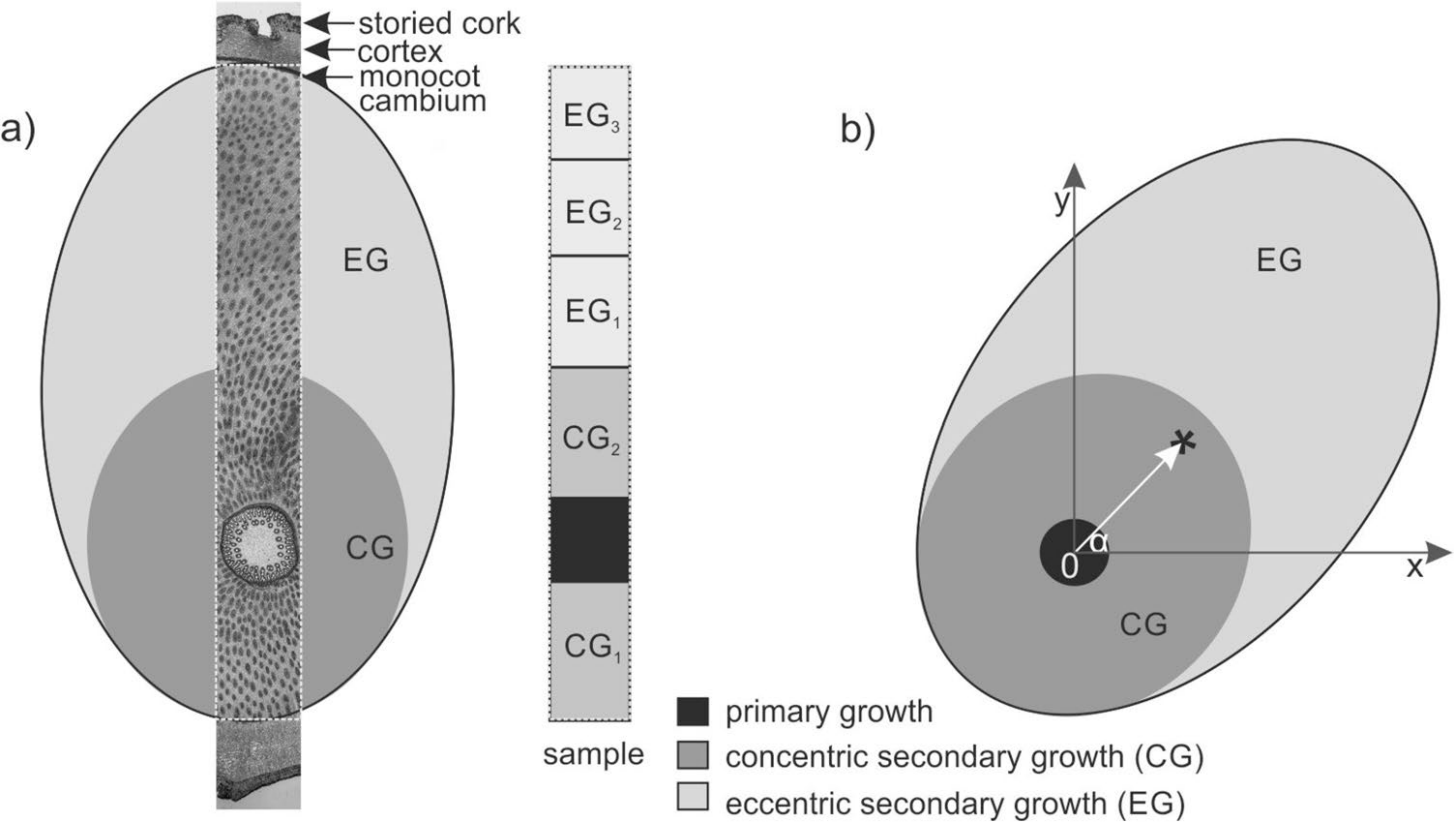
Sampling of *D. draco* roots for quantitative analysis and measurement of the eccentricity vector. **a** Scheme of the root cross section with the area indicating the location of the sample used for analyzing the radial variation of the secondary growth parameters (rectangle). The sample, covering the secondary growth along the diameter of the root, was divided into zones (CG_1_-EG_3_) for measurements, as shown in the scheme. **b** Diagram showing the measurement of the eccentricity vector (white arrow) in the root. The asterisk marks the geometric center of mass; x-axis parallel to the ground with (0) in the center of the primary growth area (actual center of the root). The distance between 0 and the asterisk represents the vector length. α—corresponds to the direction of the eccentricity vector

Successive root disks were mounted on a rotary stage and the cross section illuminated with diffused light for photography according to the procedure described in detail by Marcinkiewicz and Jura-Morawiec (2024). The material was preserved in glycerol:ethanol [1:1; v/v]. Subsequently, the surface of some root discs (6.1–8.1 cm in diameter) was smoothed using a microtome (WSL, Birmensdorf, Switzerland), sampled as described in Fig. 2a, and then sectioned into 60 μm-thick sections, with the same microtome. The unstained sections were observed with fluorescence microscopes Zeiss Axio.Lab1 (UV, 365 nm). Next, the sections were stained with a mixture of Safranin 0 and Astra Blue [1:1; v/v], dehydrated through a graded ethanol series (50–100%) and mounted in Euparal (Roth). Images of these sections were taken using a Nikon telecentric optical system 2x/0.09 and an Olympus BX41 microscope.

### Measurements

Using the images of the root disks, the eccentricity vector was measured in ImageJ (Schneider et al. 2012) employing the methods of Di Iorio et al. (2007) and Wrońska-Wałach et al. (2015), modified as described in Fig. 2b. Within each cross section the eccentricity vector was determined by measuring its length and direction. To check whether there are anatomic differences in the vascular bundles formed in CG and EG, each root was divided into five zones along root diameter CG_1_-EG_3_ (Fig. 2a.) The following parameters were measured: vascular bundle density, vascular bundle fraction, vascular bundle size, tracheid lumen fraction and vascular bundle shape. Vascular bundle density was determined by counting every vascular bundle in each particular root zone, and dividing the result by the total root zone area,  using ImageJ “count particles” function. The vascular bundle fraction was determined by counting the total number of pixels occupied by vascular bundles, and the total number of pixels occupied by parenchyma cells in each examined root zone., This was possible due to preformed staining and image thresholding. Average vascular bundle size and vascular bundle shape were measured from five randomly selected microscopic fields (each 1 mm^2^) per root zone (CG_1_-EG_3_, Fig. 2a). The vascular bundle size was determined using threshold masking and the measure particles function in the ImageJ (Schneider et al. 2012). Tracheid lumen fraction was calculated by measuring the total area of all vascular bundles in the zone (excluding phloem), and the total area of the tracheid lumen, calculating percentage, describes tracheid wall thickness. The shape, as an aspect ratio, was determined by dividing the maximum height of the vascular bundle by the maximum width. The mode of vascular bundle formation was used as a proxy to infer regions of increased cambial activity, based on previous studies of the *D. draco* stem (Jura-Morawiec 2015).

## Results

### Longitudinal variability of secondary growth

**Fig. 3:**
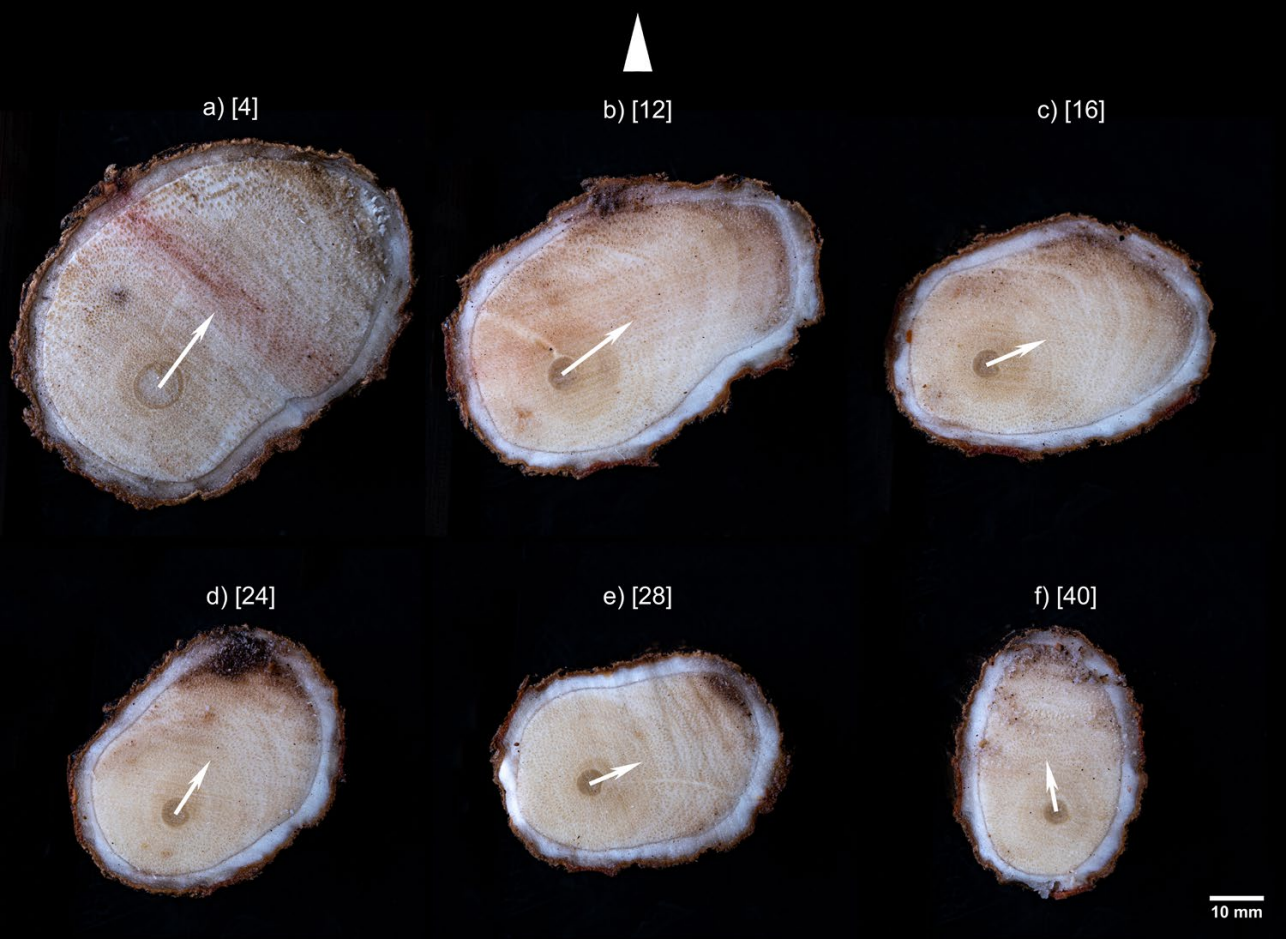
Distribution of secondary growth along the 50 cm root of *D. draco*. **a-f** Successive cross-sections of root disks, values in brackets are distances [cm] of each section from the stem; the triangle at the top of the figure indicates the topologic direction upwards, i.e., toward the surface of the ground. White arrow represents the eccentricity vector

**Fig. 4:**
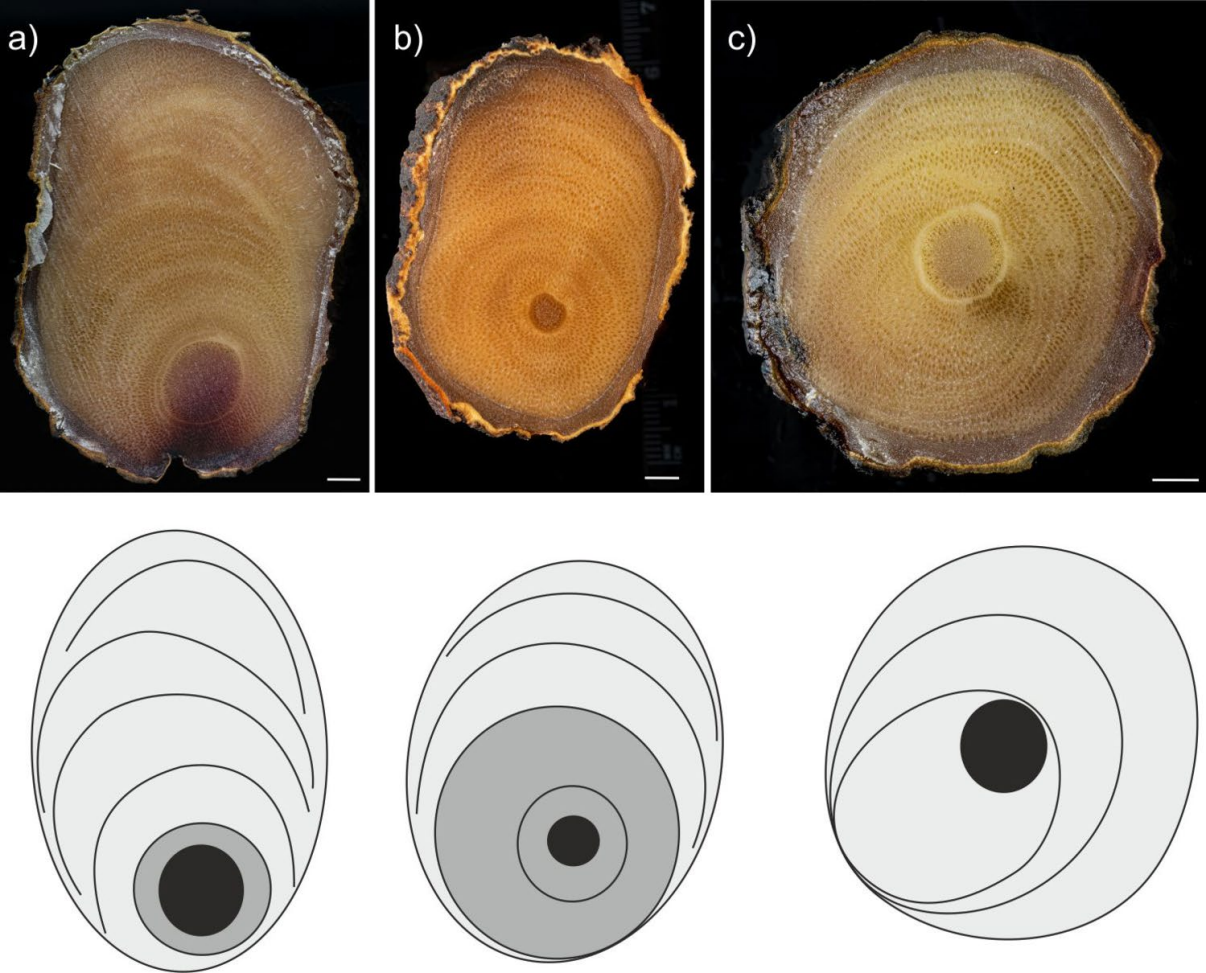
Variation in secondary growth patterns within the roots of *D. draco*. **a–c** Cross sections of exemplary root disks with diagrams of asymmetric secondary growth below, color key as in Fig. 2. Note the variable arrangement of the rings or arcs. Scale bar = 5 mm

Next to the stem, in the zone of rapid taper of the root, the eccentricity was directed upwards, with 2.3–8.3 times more growth on the upper side than on the lower side (Fig. 3a). With increasing distance from the root base, the direction of the eccentricity changed. For example, at first it was directed to the right (Fig. 3b-c), then it underwent a counterclockwise shift (Fig. 3d), and later it moved back and forth (Fig. 3e–f). In a 50 cm long root, the eccentricity varied between 23.8 and 97.5°. The EC of the root was usually associated with its oval cross section (Figs. 3, 4a, b), but in the round root with primary vascular tissue in the center (lack of eccentricity), secondary growth was also irregular, and restricted to certain sectors of the monocot cambium (Fig. 4c).

### Cross-sectional variability of secondary growth

**Fig. 5:**
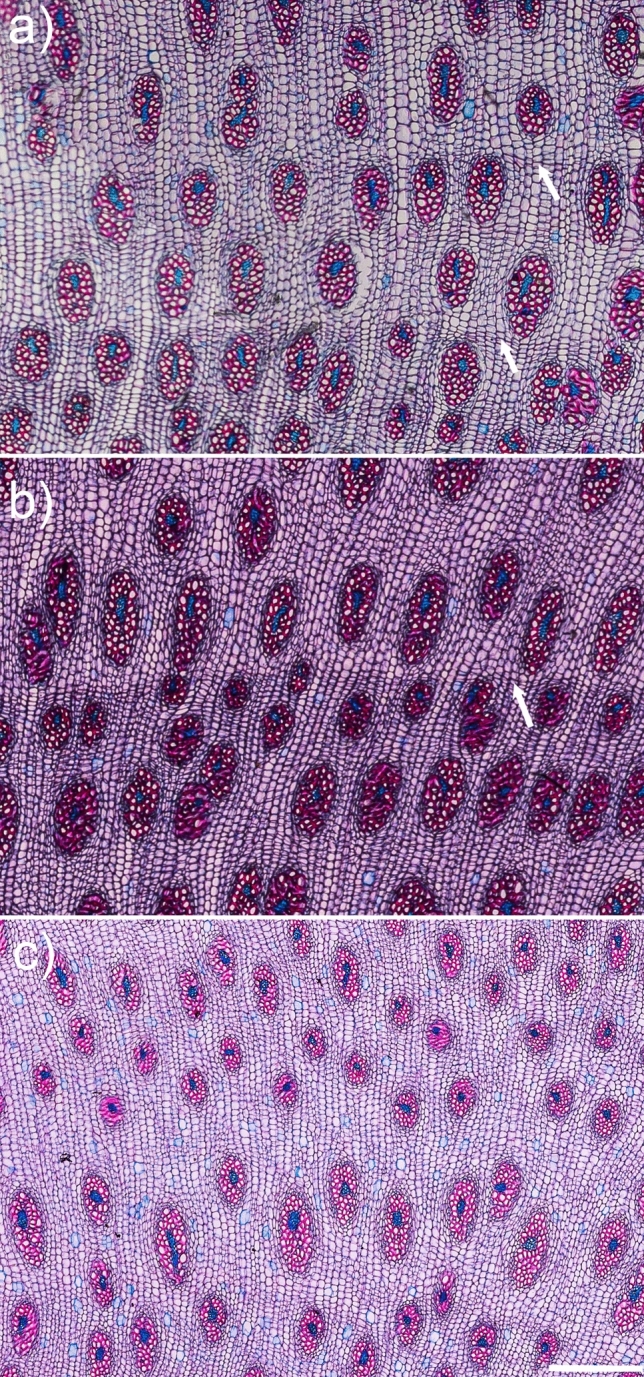
Three types of structure that can result in a ring/arc appearance in secondary growth. It may be related to differences in ground parenchyma cell wall thickness (**a**, arrows), parenchyma cell wall thickness and vascular bundle size (arrow, **b**), and vascular bundle size (with smaller bundles at the top of the image, **c**). All images are on the same scale, with a scale bar of 500 μm

The boundary between CG and EG was not sharp, but a CG was usually a more compact area with rings of vascular bundles (Fig. 4a, b). In contrast, EG showed arcs of vascular bundles stacked on top of each other (Fig. 4a, b). Distinct rings or arcs were the result of varying thickness of the ground parenchyma cell walls, variation in bundle size, or a combination of both (Fig. 5). It is worth noting that stable and gradual changes in bundle density, shape and size were observed in CG (Table 1). Conversely, EG sometimes showed sharp changes in these parameters resulting in characteristic arcs in sections. Thus, in this case, averaged values (Table 1) may not clearly describe what is readily visible in macroscopic (Fig. 4) and microscopic (Fig. 5) examination.

**Table 1:** Quantitative analysis of the anatomic characteristics of the concentric secondary growth (CG_1_**–**CG_2_) and eccentric secondary growth (EG_1_**–**EG_3_) in the stem-borne roots of four *D. draco* individuals (sample1**–**4)

Root sample	Root zone	Vascular bundle density [No./mm^2^]	Vascular bundle fraction [%]	Average vascular bundle size min-avg-max [mm^2^]	Tracheid lumen area fraction [% of vb]	Vascular bundle shape aspect ratio [height/width]
Sample1	CG_1_	3.29	30	0.056–0.090–0.140	22	2.23
	CG_2_	3.82	28	0.059–0.082–0.124	22	2.45
	EG_1_	2.71	24	0.056–0.080–0.107	23	1.65
	EG_2_	1.89	28	0.048–0.087–0.137	31	2.05
	EG_3_	2.12	32	0.055–0.090–0.139	36	1.83
Sample2	CG_1_	4.32	41	0.044–0.060–0.080	16	2.5
	CG_2_	4.20	27	0.060–0.075–0.087	15	2.5
	EG_1_	2.24	28	0.046–0.093–0.151	33	2.05
	EG_2_	1.61	21	0.064–0.101–0.149	45	1.66
	EG_3_	2.79	22	0.053–0.077–0.100	39	2.2
Sample3	CG_1_	4.32	39	0.089–0.114–0.151	25	2.26
	CG_2_	3.83	37	0.081–0.129–0.166	18	2.55
	EG_1_	3.66	38	0.085–0.123–0.195	20	2.02
	EG_2_	3.44	27	0.077–0.090–0.135	26	1.77
	EG_3_	2.55	21	0.094–0.151–0.178	25	1.62
Sample4	CG_1_	3.13	39	0.076–0.111–0.171	27	2.65
	CG_2_	3.41	27	0.080–0.112–0.170	25	2.82
	EG_1_	3.66	23	0.060–0.099–0.119	23	1.84
	EG_2_	3.44	33	0.081–0.123–0.175	27	2.26
	EG_3_	2.55	22	0.071–0.134–0.217	29	2.23

**Fig. 6:**
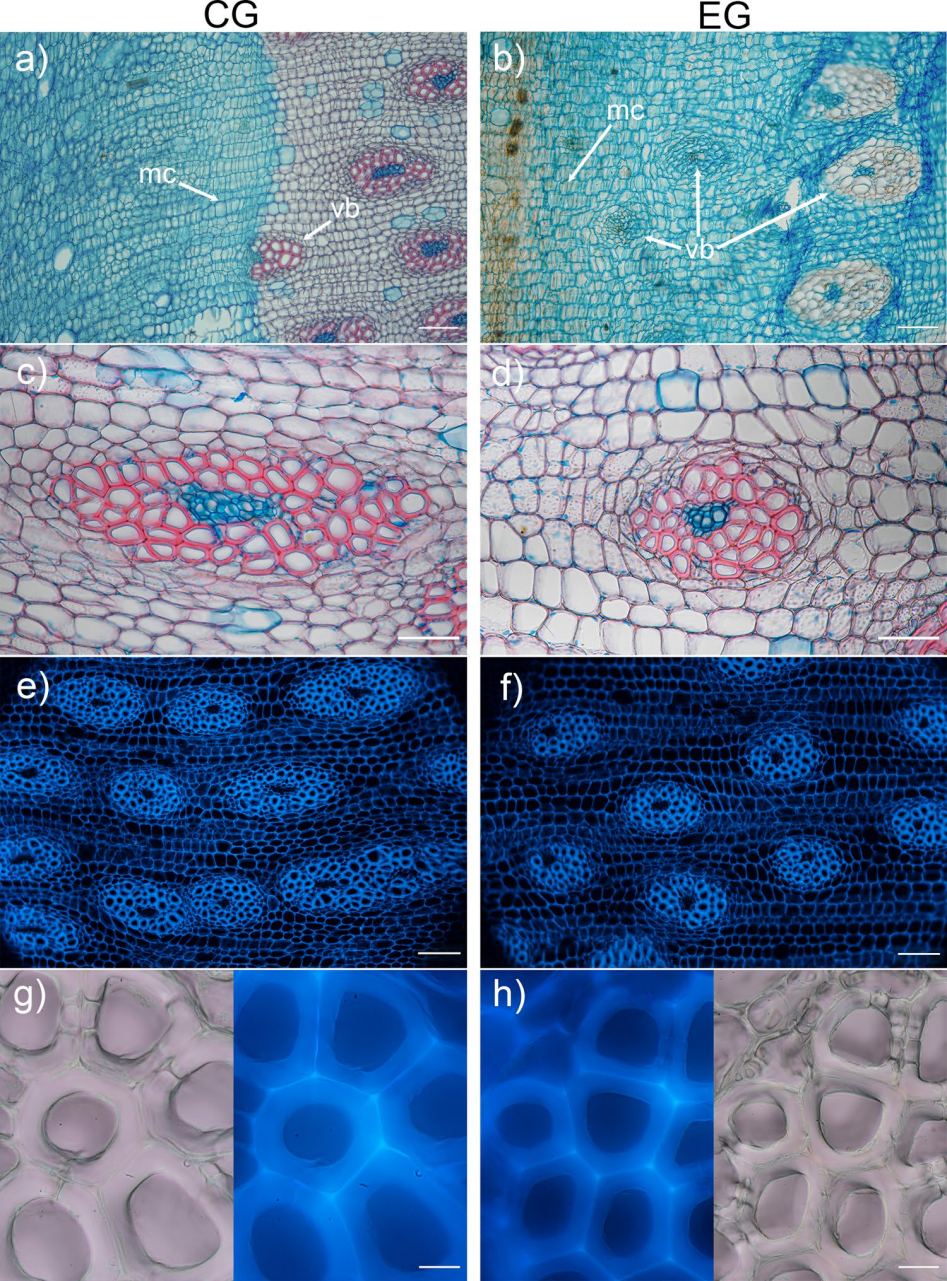
Anatomic details of the concentric secondary growth (CG) on the left and eccentric secondary growth (EG) on the right in the transverse sections of a *D. draco* root. **a–b** Brightfield microscopy, with Safranin 0 and Astra blue staining. Note different modes of vascular bundle development indicating the activity of the monocot cambium. **c–d** Brightfield microscopy, with Safranin 0 and Astra blue staining displaying variability in vascular bundle size and shape. **e–f** Autofluorescence (UV 365 nm) showing difference in density, size and shape of vascular bundles. **g–h** Autofluorescence (UV 365 nm) and bright-field images of the same fragment reveal the absence of thickened tracheid walls (compression wood) and a gelatinous G-layer (tension wood), indicating a lack of reaction wood in both CG and EG. mc, monocot cambium; vb, vascular bundle; scale bars = 200 μm (**a–b**, **e–f**), 100 μm (**c–d**), 20 μm (**g–h**)

The sectors of monocot cambium adjacent to zones CG_1_ and EG_3_ differed in their activity. The monocot cambium was more active near zone EG_3_, as shown by the mode of vascular bundle formation (Fig. 6a, b). The CG and the EG differed in vascular bundle density and size but also in the proportion of their components i.e., tracheid lumen area fraction and tracheid wall area fraction (Table 1). Namely, the CG was characterized by a higher vascular bundle density, with a lower tracheid lumen area fraction and thus a higher tracheid wall area fraction. The differences between CG and EG were also related to the shape and arrangement of the vascular bundles. Along the root diameter, various shapes could be easily distinguished from elongated to nearly circular (Fig. 6c–d; Table 1). In CG, the vascular bundles were usually elongated (Fig. 6c and Table 1). In EG, they were variously shaped, including nearly oval-shaped bundles (Fig. 6d), which were arranged in lines according to  their similar shape and size (Fig. 5b, c). No specific structural features characteristic of reaction wood were identified (Fig. 6g–h).

## Discussion

Secondary growth in the roots is responsible for storage and transport of water, minerals and other resources. In addition, it also allows the arborescent plant to withstand the swaying of the trunk and prevent it from falling over, which is one of its most important adaptive traits. In monocotyledons of the genus *Dracaena,* such as *D. draco*, roots increase in diameter through secondary growth (Tomlinson and Zimmermann 1969; Kauff et al. 2000; Carlquist 2012). In this study, we found that monocot cambium activity is highly variable around its circumference and along the roots of *D. draco*, altering their structure, shape and size. The distribution of EG suggests that the activity of the monocot cambium in the roots is affected by more dynamic factors than in the stem (Fisher 1975), and that eccentric growth is not associated with reaction wood characteristics.

### Longitudinal variability of secondary growth

Marcinkiewicz and Jura-Morawiec (2024) noted that secondary growth at the stem–root junction is eccentric and probably affects the stabilization of the stiff aboveground part of the plant. Our study adds to this knowledge by analyzing the distribution of secondary growth as it moves away from the root base toward the first ramification, in plants grown in the greenhouse and in the field. The considerable variation in the direction of the eccentricity vector along the root and, contrary to our assumption, the fact that secondary growth is not always initially concentric, suggest that the monocot cambium is subjected to different dominant factors along root length (mechanical, environmental, hormonal). As dragon trees are long-lived, branched and need to maintain the heavy, water-storing aboveground organs (Jura-Morawiec and Marcinkiewicz 2020), their root systems need to be well adapted to withstand static loads caused by gravity, and dynamic loads made by wind in order to prevent uprooting (reviewed by Stubbs et al. 2019). Static loads increase slowly with plant size (Niklas and Spatz 2000), while dynamic loads can change rapidly, from one hour to the next (Gardiner et al. 2016). Thus, it is likely that eccentric secondary growth is related to the adaptation of root anatomy and morphology to changes in self-loading related to plant size and water content, interacting with wind exposure, in the case of plants growing outdoors.

Apart from the factors resulting from crown–stem–root interactions, variable activity of the monocot cambium along the root may result from root-soil stresses due to soil pressure, growth around various obstacles such as other roots and stones. Furthermore, if part of the root system is exposed, as is sometimes the case with the shallow root system of dragon trees, access to light or the release from soil resistance may also increase the activity of the monocot cambium. In addition to external factors influencing secondary growth, internal hormonal regulation also controls cambial activity (reviewed by Strock and Lynch 2020). Fisher (1975) noted a relationship between enhanced monocot cambium activity and high auxin levels on the lower side of a leaning monocot stem. However, the experimental quantification of the effect of a single external or internal factor on monocot cambium activity in roots is still a challenging task.

### Cross-sectional variability of secondary growth

Previous studies on eccentric secondary growth in monocots have shown that increased activity of the monocot cambium can occur in the lower sectors of the cambial circumference of the stem (Fisher 1975) and branch (Krawczyszyn and Krawczyszyn 2016)., At the base of the root, it tends to be more pronounced in the upper sectors of cambial circumference (Marcinkiewicz and Jura-Morawiec 2024). We have shown that the local increase in monocot cambium activity can vary, relating to sectors of the circumference of stem-borne roots of *D. draco* in an angular range between 0° and 180°. Interestingly, nearly round root parts of *D. draco* can still indicate highly asymmetric secondary growth (Fig. 4c).

Variable distribution of secondary growth along the roots was associated with differences at the anatomic level. It is known that areas characterized by substantial secondary growth differ in vascular bundles size and density (Fisher 1975; Krawczyszyn and Krawczyszyn 2016). Our study represents a different approach, and apart from bundle size and density, showed the variation along the root in the proportions of functionally distinct secondary growth components (vascular bundle fraction, tracheid wall, tracheid lumen). The revealed differences in anatomic characteristics may indicate a spatial functional separation of the mechanical, transport and storage functions in the root. EG, with a higher tracheid lumen area and a lower vascular bundle fraction (higher parenchyma fraction), suggests a primary role in water transport and storage. Conversely, CG values indicate a stronger specialization for mechanical support. Importantly, however, secondary growth in roots was not accompanied by structural changes in the tracheids that could indicate the presence of reaction wood. None of the criteria outlined by Donaldson and Singh (2016) for identifying reaction wood, such as rounded or abnormally thickened tracheid walls, , intercellular spaces at tracheid corners, or the presence of a gelatinous (G) layer, were observed. Our results are in line with previous studies that reported the absence of reaction wood characteristics in tracheids of eccentric secondary growth in monocot stem and branch (Fisher 1975; Krawczyszyn and Krawczyszyn 2016).

## Conclusion

Our study is a pioneering effort to investigate the variability of secondary growth along roots in monocotyledons. We have shown that the activity of the monocot cambium varies greatly around the meristem circumference and along the root axis, resulting in an irregular distribution of its derivatives. The eccentric distribution of secondary growth and the ring/arc pattern in monocot roots is more variable than that recorded for the monocot stem (Fisher 1975) and branch (Krawczyszyn and Krawczyszyn 2016). The variation in the proportions of secondary growth components (vascular bundles, tracheid wall, tracheid lumen) indicates that some regions could be primarily involved in water transport and storage, whereas others, are more specialized for mechanical support, but with no reaction wood characteristics.

## Data Availability

The datasets generated during and/or analyzed during the current study are available from the corresponding author on reasonable request.

## Abbreviations

CG: Concentric secondary growth
EG: Eccentric secondary growth
